# Community-Led, Cross-Sector Partnership of Housing and Health Care to Promote Aging in Place (Unite Health Project): Protocol for a Prospective Observational Study

**DOI:** 10.2196/47855

**Published:** 2023-06-29

**Authors:** Lesli Skolarus, Erica Thrash-Sall, Abby Katherine Hellem, Michael Giacalone Jr, James Burke, Chun Chieh Lin, Sarah Bailey, Casey Corches, Mackenzie Dinh, Amanda Casetti, Maria Mansour, Kaitlyn Bowie, Rylyn Roth, Candace Whitfield, Anne Sales

**Affiliations:** 1 Davee Department of Neurology Northwestern University Chicago, IL United States; 2 Presbyterian Villages of Michigan Flint, MI United States; 3 Department of Neurology Michigan Medicine University of Michigan Ann Arbor, MI United States; 4 Hamilton Community Health Network Flint, MI United States; 5 Department of Neurology The Ohio State University Columbus, OH United States; 6 Bridges Into the Future Flint, MI United States; 7 Emergency Medicine Michigan Medicine University of Michigan Ann Arbor, MI United States; 8 Sinclair School of Nursing University of Missouri Columbia, MO United States; 9 Department of Family and Community Medicine University of Missouri Columbia, MO United States

**Keywords:** aging in place, stroke prevention, hypertension, blood pressure, social determinants of health, affordable housing, older adults, community-based participatory research, implementation evaluation

## Abstract

**Background:**

For many older Americans, aging in place is their preferred living arrangement. Minoritized and socioeconomically disadvantaged older adults are up to 3 times more likely to experience disability than other groups, which increases their likelihood of being unable to age in place. Bold ideas to facilitate aging in place, particularly among vulnerable populations, are needed. One such idea is the Unite care model, a community-initiated, academic-supported, cross-sector initiative that combines 2 sectors: housing and health care. The Unite care model colocates a federally qualified health center clinic on an older adult affordable housing campus in Flint, Michigan.

**Objective:**

There are two aims to this study. Aim 1 is to evaluate the implementation of the Unite care model in terms of acceptability, adoption, and penetration. Aim 2 is to determine which older adults use the care model and whether the care model promotes aging in place through risk factor reduction and improvement in the physical and social environment.

**Methods:**

We will assess the care model using a concurrent, exploratory mixed methods design. For aim 1, acceptability will be assessed through semistructured interviews with key stakeholder groups; adoption and penetration will be assessed using housing and health care records. For aim 2, residents residing in the Unite clinic building will participate in structured outcome assessments at 6 and 12 months. Risk factor reduction will be measured by change in systolic blood pressure from baseline to 12 months and change in the physical and social environment (item counts) will also be assessed from baseline to 12 months.

**Results:**

Data collection for aim 1 began in July 2021 and is anticipated to end in April 2023. Data collection for aim 2 began in June 2021 and concluded in November 2022. Data analysis for aim 1 is anticipated to begin in the summer of 2023 and analysis for aim 2 will begin in the spring of 2023.

**Conclusions:**

If successful, the Unite care model could serve as a new care model to promote aging in place among older adults living in poverty and older Black Americans. The results of this proposal will inform whether larger scale testing of this new model of care is warranted.

**International Registered Report Identifier (IRRID):**

DERR1-10.2196/47855

## Introduction

### Background

Aging in place, defined as the ability to stay in one’s current residence by securing necessary support in response to evolving needs, is overwhelmingly preferred among older adults [1-3]. Aging in place also provides health benefits for older adults [4], via sustaining established social networks and care services [5,6]. Furthermore, aging in place is overwhelmingly favored by policy makers because it may delay or avoid the costly option of institutional care; 1 year of nursing home care costs about US $80,000 [7]. For many older Americans, aging in place is their only option as assisted living facilities require significant out-of-pocket spending. Thus, strategies to support aging in place are critical, particularly among low-income Americans.

Disability is the strongest predictor of nursing home admissions [8]. Over 25% of older adults experience activity limitations and cognitive impairment [9-11]. Disability disproportionately impacts Black people and low-income Americans and negatively impacts aging in place. Minority and socioeconomically disadvantaged older adult populations are up to 3 times more likely than other groups to experience disability [12-14]. Furthermore, Black people have the highest incidence of Alzheimer disease and Alzheimer disease–related dementias (AD/ADRDs) with a 2-fold greater incidence compared with White people [15-19].

Efforts to reduce disability and optimize the environment may help facilitate aging in place. Hypertension is the most important modifiable cardiovascular risk factor [20-24] and a predictor of nursing home admission [8]. Hypertension treatment reduces the incidence of stroke [25] and may reduce the incidence of AD/ADRDs [26], which are the leading causes of disability and strongest predictors for not aging in place [27-29]. Therefore, hypertension treatment, complemented by physical and social environment optimization, may promote aging in place by reducing informal caregiving needs, unmet needs, falls, and disability [30-33].

### Unite Care Model

The Unite care model is a community-initiated and led, academically supported, cross-sector initiative that combines housing and health care to promote aging in place by addressing personal needs and optimizing the physical and social environment. The Unite care model includes a federally qualified health center clinic on an older adult affordable housing campus in Flint, Michigan, home to over 700 predominately Black older adults. The Unite clinic is located within the largest older adult congregate housing building, home to about 300 older adults.

**Figure 1:**
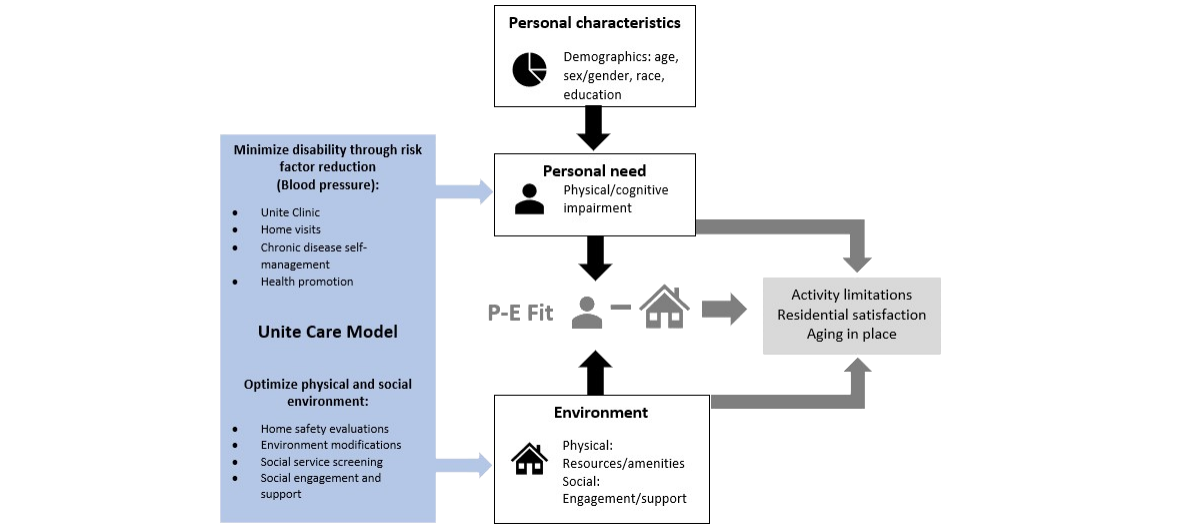
Unite care model.

The Unite care model is comprised of 3 components: Colocation of a federally qualified health center clinic on the affordable housing campus, home medical visits, and community health worker–supported care. The Unite care model will promote aging in place by optimizing (1) medical care including onsite clinic and home visits; (2) the social environment through clinic referrals for home safety evaluations and facilitating environmental modifications; and (3) the social environment by community health worker–led social engagement and support. Figure 1 depicts this care model. Overall, the Unite care model flips the paradigm that asks the most vulnerable older adults to seek out the medical safety net. Instead, the Unite care model will bring the safety net to older adults to support aging in place.

The Unite care model builds on Community Aging in Place—Advancing Better Living for Elders (CAPABLE), a randomized trial of home occupational therapy and nursing sessions, and home repairs to reduce disability and promote aging in place through physical environmental optimization compared to controls. The CAPABLE group experienced a 30% (4.00 to 2.22) decrease in disability scores compared to controls at 5 months but was not sustained at 1 year [34]. Booster visits, social service screening, and community health worker support were identified by the research team and other researchers as mechanisms that could improve sustainability [34,35]. The Unite care model builds on the CAPABLE trial by expanding the intervention to provide medical care to reduce disability risk factors and community health workers to optimize the social environment.

### Aims and Objectives

This study will evaluate the Unite care model through 2 aims.

- Aim 1: To assess the implementation and impact of the Unite care model in terms of acceptability, adoption, and penetration among key stakeholder groups using an exploratory mixed methods design
- Aim 2: To determine which older adult residents who reside in the Unite clinic building use the Unite care model and whether the model promotes aging in place through cardiovascular risk factor reduction and improvement in the physical and social environment

## Methods

### Study Design

A concurrent exploratory mixed methods approach will be used to evaluate the implementation of the Unite care model.

### Ethics Approval

This study is approved by the University of Michigan Institutional Review Board (HUM00197049).

### Study Setting

Flint, Michigan is a community with a large burden of chronic disease and poverty. Flint is recovering from the water crisis, where the city’s drinking water was contaminated with excessive levels of lead for over 18 months. Flint has a population of about 80,000, 57% of which are Black; 40% live in poverty and is ranked 80 out of 83 Michigan counties on Robert Wood Johnson Foundation County Health Rankings [36].

The Unite care model takes place at a heavily charity-subsidized older adult (55 years and older) affordable apartment complex in Flint, Michigan. The apartment complex is situated within a broader, informal, older adult housing campus. Within a 3-square mile radius of the apartment complex, an estimated 700 independently living older adults reside at a public housing apartment building, a US Department of Housing and Urban Development senior living building, and single-family homes. About 75% of the older adults are Black, many earning less than US $12,000 annually.

### Aim 1: Assessing the Implementation and Impact of the Unite Care Model

#### Overview

We will determine how much and for whom (ie, adoption and penetration) and how well (ie, acceptability) the Unite care model is implemented. These implementation outcomes are necessary preconditions to understand the effectiveness of the Unite care model [37].

#### Acceptability

To measure the acceptability of the Unite care model, stakeholders from three different groups: (1) housing and health care administrators, (2) providers, and (3) older adult residents will be recruited to participate in semistructured interviews based on the theoretical framework of acceptability [38], theoretical domains framework, and Proctor’s definition of acceptability [37]. Interview guide questions will explore constructs including affective attitude, burden, perceived effectiveness, ethicality, intervention coherence, opportunity costs, and self-efficacy. Interview guides will be tailored to each population, given acceptability is distinct among those who received the care model compared to those who deliver the care model [38]. Sample interview guide questions for each group are shown in Table 1.

Housing and health care administrators (n=9) will be interviewed at the time of the clinic opening (baseline) and again at 18 months. Snowball sampling will be used to identify all key administrators. Providers (n=5) will be interviewed at 12 months and will include Unite clinic staff (nurse practitioner and medical assistant) and housing employees who provide wraparound services to residents (resident wellness director, resident service coordinator, and community health worker). Older adult residents (n=36) will also be interviewed at 12 months. Older adult positive and negative opinion leaders will be purposely sampled across three subgroups: (1) older adult residents who use the clinic (n=12), (2) older adult residents who have attempted to use the clinic (n=12), and (3) older adult residents who do not use the clinic (n=12). We will identify older adults pertaining to each subgroup based on structured survey responses (aim 2) and notes kept by the research team. Interviews will be conducted in person as possible at a location convenient to the participant, with the alternative of video or telephone calls given the individual’s availability, preference, and COVID-19 restrictions. Participants will receive US $40.

Semistructured interviews will be audio recorded, professionally transcribed, and reviewed by the research team for accuracy. We will use ATLAS.ti (ATLAS.ti Scientific Software Development GmbH), a qualitative data analysis and research software, to facilitate qualitative analysis. We will perform direct content analysis using the theoretical framework of acceptability and theoretical domains framework as a template. Two members of the research team will independently code each transcript, compare their coding, discuss discrepancies, and agree on final codes. Several strategies will be used to enhance the reliability and validity of the analysis including member checks, peer debriefing, and concurrent data collection and analysis described below.

**Table 1:** Overview of aim 1 interviews.

Population	Participants, n	Timeline	Sample questions
Housing and health care administrators	9	Baseline (clinic opening) 18 months	Now, as the Unite clinic is about to open, how are you feeling about participating in this project? (affective attitude) How has the clinic impacted your daily workload? (burden and barriers)
Providers	5	12 months	How are things going at the clinic? (positive and negative affect) What do residents say about the clinic? (social norms)
Older adults	36	12 months	How do you feel about the clinic? or What do your friends think about the clinic? (positive/negative affect) What do you think would happen if you went to the clinic? (outcome expectations)

#### Adoption

Adoption, defined as the Unite care model uptake by older adult residents, will be quantified by the number of provider home visits and community health worker or resident service coordinator interactions. The number of home visits will be self-reported. Community health worker or residence service coordinator interactions will be sampled daily for 1 week per month via a Qualtrics survey completed by the community health worker. The survey will assess the number of residents served that day as well as the services provided to each resident.

#### Penetration

Penetration is defined as the proportion of people who participate in a given program. We will explore the role of clinic proximity as a predictor of Unite care model usage. The research team will obtain data from the Unite clinic electronic health record, including patient-level data (addresses, demographics, and comorbidities). Patient addresses will be geocoded using Google’s interface to identify if the patient resides on the older adult affordable housing campus and specifically in the Unite clinic building.

The primary outcome is the proportion of older adults who reside on the older adult affordable housing campus who are cared for by the Unite clinic. The research team will make 2 types of comparisons between older adults who reside in the Unite clinic building compared to older adults who reside on the campus but not in the Unite clinic building. First, for measures that can only be defined in aggregate (ie, number of new patient visits), the research team will compare mean per-patient usage measures between populations. Specifically, we will compare the mean of each usage variable divided by the number of patients living in each setting using *t* tests and the proportion of new and return visits using chi-square tests across settings. Second, for measures where individual-level data can define the population at risk (number of missed appointments and appointment timeliness), we will fit appropriate regression models to predict the usage measures (dependent variable) accounting for residence (Unite clinic building vs other older adult campus) and demographics known to predict missed appointments (age, race, and sex), comorbidities and dual eligibility (independent variables).

#### Integration of Qualitative and Quantitative

The qualitative (acceptability) and quantitative data (adoption and penetration) will be collected and analyzed during the same time. Integration will merge the qualitative and quantitative data after their respective analyses are complete [39]. We will merge the data through narrative integration, whereby the qualitative results are presented followed by the quantitative results and jointly displayed in a table. Conceptually, the research team believes that the Unite care model must be found to be acceptable by stakeholders as a precursor to adoption and penetration. Thus, the qualitative data will be presented first. The research team will also assess the concordance of the data integration. If discordance is identified, additional data may be gathered.

### Aim 2: Determining Which Older Adults Residing in the Unite Clinic Building Use the Unite Care Model and Whether the Model Promotes Aging in Place

#### Overview

We seek to understand the usage of the Unite care model and to assess the impact of the components of the person-environment fit model, hypertension as a predictor of disability, and the environment. This is a prospective observational study with baseline, during, and post-Unite care model initiation assessments.

### Study Population Recruitment and Enrollment

Residents of the Unite clinic building (n=300) will be recruited to complete in person, prospective observational assessments in which they will be asked structured interview questions and have their blood pressure taken. Residents will be recruited from building central locations, including community and recreation rooms, in addition to fliers, word-of-mouth, and door-to-door knocking. Recruitment will continue until all residents have been contacted and provided the opportunity to consent or decline. The housing staff will aid the research team in contacting hard-to-reach residents. Residents who do not respond to the research team and housing staff after multiple contact attempts will be considered a decline. We will use our ongoing successful retention strategies, which include maintenance of contact and obtaining alternate contact information, including those of family members, in the event of change in contact information. The assessments will take place in a private space in the Unite clinic building. Participants will be given US $20 at baseline and US $25 for each outcome assessment.

Baseline assessments will begin at the time of the Unite clinic opening, with 6- and 12-month outcome assessments. The assessments will measure resident cognition, activity limitations [40-42], blood pressure, Unite clinic usage, physical [43] and social environment [44], health-related social needs [45], and housing transition information. Blood pressure will be measured by research staff using the OMRON 7 series upper arm blood pressure monitor (OMRON Healthcare, Inc) in accordance with national standards for measurement. Housing status transition will be obtained from the Unite clinic building records including 2 years prior to the start of the Unite care model. Participant responses and blood pressure measurements will be stored in a Research Electronic Data Capture database (Vanderbilt University). Table 2 depicts which measures are assessed at each outcome assessment.

**Table 2:** Comparison of aim 2 measures by outcome assessment.

	Baseline	6 months	12 months
Sociodemographics	✓		
Comorbidities	✓		
Self-rated health	✓		✓
Activity limitations	✓		
Cognitive impairment	✓		
Unite care model usage		✓	✓
Blood pressure measurement	✓	✓	✓
Physical environment	✓		✓
Social environment	✓		✓
Housing status	✓	✓	✓

### Data Analysis

The primary analysis is the change in systolic blood pressure (SBP) from baseline to 12 months. Meta-analyses have found that for a given blood pressure, SBP predicted over 90% of the risk of ischemic heart disease and stroke and that a 2 mm Hg decrease in SBP could reduce stroke and ischemic heart disease mortality [46]. Analysis will fit a linear regression model with the outcome of 12-month SBP, including baseline SBP and a binary predictor of Unite care model patient (yes vs no) as independent variables.

Other secondary analysis end points will include determining whether functional and cognitive limitations are associated with care model usage, change in physical and social environment, and moves out of the Unite clinic building. To determine if functional and cognitive limitations, including activity limitations and AD/ADRD are associated with Unite care model usage, the research team will build a logistic regression model predicting Unite utilization, including age, sex, race, ethnicity, AD/ADRD status, level of functional disability, self-reported health, and baseline hypertension as independent variables. Change in physical and social environment (item counts) will be assessed using a Poisson regression with an outcome of 12-month values, adjusting for baseline values and including an intervention indicator. Moves out of the clinic building will be assessed by comparing moves out of the building in the 2 years prior to the Unite care model compared to year 2 of the Unite care model.

Of the 300 older adults residing in the Unite building, we estimate that 5% will elect not to enroll, resulting in a study population of 285 older adults. We estimate that we will have 89% power to detect a 2-point change in SBP within older adults assuming a mean baseline SBP of 162 mm Hg and SE of change in SBP over time of 10 mm Hg, based on prior data in this community and community studies [47,48], and a 10% loss to follow-up using an analogous test to our primary analysis. If loss to follow-up increases to 20%, power to detect a 2-point change in SBP would decrease to 85%. The effect may be larger. We will have 100% power to detect a 4-point SBP change if loss to follow-up were 20%.

### Overall Assessment of Unite Care Model

After executing these aims, true to our community-engaged approach, our advisory board comprised of older adults, Unite partners (housing and health care), insurance, and local government stakeholders will review the results. Our evaluation of the care model will be holistic and require considerable judgment as no single metric nor combination of metrics is likely to capture the complexities of the initial Unite experience. The panel will then judge whether the Unite care model is sufficiently promising to justify efforts to develop similar models of care.

## Results

This study was funded by the National Institutes of Health-National Institutes on Aging in April 2021 and received institutional review board approval from the University of Michigan in May 2021. Semistructured interviews for aim 1 began in July 2021 and are anticipated to end in April 2023, with an anticipated total 45 stakeholders, including administrators, providers, and older adult residents. Structured interviews for aim 2 began in June 2021 and ended in November 2022. For aim 2, at baseline, 265 residents were approached, of which 179 residents enrolled. In total, 149 residents completed 6-month outcomes and 131 residents completed the final 12-month outcome. Data analysis for aim 1 is anticipated to begin in summer 2023; analysis for aim 2 will begin in spring 2023.

## Discussion

### Anticipated Findings

We anticipate the following outcomes: (1) among older adults residing in the Unite clinic building, those with functional and cognitive limitations (ie, activity limitations, AD/ADRD) will be more likely to use the Unite care model than those without limitations; (2) among older adults residing in the Unite clinic building, those who receive care through the Unite care model will have reduced SBP compared to those who do not receive care through the Unite care model; and (3) Among older adults residing in the Unite clinic building, those who receive care through the Unite care model will have an increase in physical environment modifications and a decrease in social isolation compared to those who do not receive care through the care model.

### Strengths and Limitations

The evaluation of the Unite care model is at the intersection of community engagement and implementation science. The Unite care model is a community-led and community-implemented intervention where the academic partners are supporting the initiative by conducting the analysis. Cross-sector collaborations such as Unite where housing and health care are partnering is 1 approach to addressing adverse social determinants of health. Often, investment in 1 sector accrues benefits in another sector while increasing the costs and resource usage in the investing sector [49-51]. In this case, the Unite care model is initiated and led by both housing and health care stakeholders, a cross-sector partnership promoting a shared assessment of value [52]. Multilevel interventions, such as the Unite care model, may interact to create synergistic effects, but rigorous assessments are rarely completed [53,54]. Our robust, holistic assessment of the Unite care model will inform whether larger scale testing of this new care model is warranted.

Potential challenges include recruitment and retention of participants in aim 2. We will employ our long-standing community recruitment and retention practices to reduce this barrier. Another potential pitfall lies in the study duration. The 2-year study timeframe may not be long enough to accrue outcomes of interest including aging in place and change in physical and cognitive impairment. Long-term follow-up of the Unite population could occur in subsequent research.

### Conclusions

Understanding the impact of combining older adult housing and health care will inform future cross-sector collaborations. Additionally, overlaying an implementation science-centered evaluation on a community-led initiative is an example of how academic teams can fully support their community partners.

## Abbreviations

AD: Alzheimer disease
ADRD: Alzheimer disease–related dementia
CAPABLE: Community aging in place-advancing better living for elders
SBP: systolic blood pressure
