# Correction: Community-Led, Cross-Sector Partnership of Housing and Health Care to Promote Aging in Place (Unite Health Project): Protocol for a Prospective Observational Study

**DOI:** 10.2196/54662

**Published:** 2023-11-21

**Authors:** Lesli Skolarus, Erica Thrash-Sall, Abby Katherine Hellem, Michael Giacalone Jr, James Burke, Chun Chieh Lin, Sarah Bailey, Casey Corches, Mackenzie Dinh, Amanda Casetti, Maria Mansour, Kaitlyn Bowie, Rylyn Roth, Candace Whitfield, Anne Sales

**Affiliations:** 1 Davee Department of Neurology Northwestern University Chicago, IL United States; 2 Presbyterian Villages of Michigan Flint, MI United States; 3 Department of Neurology Michigan Medicine University of Michigan Ann Arbor, MI United States; 4 Hamilton Community Health Network Flint, MI United States; 5 Department of Neurology The Ohio State University Columbus, OH United States; 6 Bridges Into the Future Flint, MI United States; 7 Emergency Medicine Michigan Medicine University of Michigan Ann Arbor, MI United States; 8 Sinclair School of Nursing University of Missouri Columbia, MO United States; 9 Department of Family and Community Medicine University of Missouri Columbia, MO United States

In “Community-Led, Cross-Sector Partnership of Housing and Health Care to Promote Aging in Place (Unite Health Project): Protocol for a Prospective Observational Study” (JMIR Res Protoc 2023;12:e47855) the authors noted one error.

Multimedia Appendix 1 was initially published as follows:

Multimedia Appendix 1. Peer review report by the Community-Level Health Promotion (CLHP) Study Section - Healthcare Delivery and Methodologies Integrated Review Group - Center for Scientific Review (National Institutes of Health, USA).

This file has been removed from the article. Accordingly, the boiler plate text was changed from:

The proposal for this study was peer-reviewed by: Community-Level Health Promotion (CLHP) Study Section - Healthcare Delivery and Methodologies Integrated Review Group - Center for Scientific Review (National Institutes of Health, USA). See the Multimedia Appendix for the peer review report;

To the following:

The proposal for this study was peer-reviewed by: Community-Level Health Promotion (CLHP) Study Section - Healthcare Delivery and Methodologies Integrated Review Group - Center for Scientific Review (National Institutes of Health, USA);

The correction will appear in the online version of the paper on the JMIR Publications website on November 21, 2023 together with the publication of this correction notice. Because this was made after submission to PubMed, PubMed Central, and other full-text repositories, the corrected article has also been resubmitted to those repositories.

